# Observations on a Peculiar Form of Bilious Fever

**Published:** 1841-02

**Authors:** John Hardin

**Affiliations:** Greensburg, Ky.


### Art. VI.—*Observations on a peculiar form of Bilious Fever.*

By John Hardin, M. D., of Greensburg, Ky.

In an essay on Congestive Fever, published in the first Number of the Louisville Journal of Medicine and Surgery, two cases were reported on account of a peculiar character-

---

istic. This peculiarity consisted in the copious evacuation from the bowels of mucus, as green as wheat, inodorous, exceedingly acid and incapable of communicating any tinge to water. Since the publication of those cases, the writer has had extensive opportunities to observe the same form of disease. The observations shall be detailed in the order in which they were made. The detail of a few cases will sufficiently develope the symptoms, course, and treatment of the disease under consideration. It will be borne in mind, that the two published cases, already referred to, were fatal in their termination; which is true of three of those that I am now about to report:

*Case 1st.* On the 13th day of April, 1838, I was called to the country to see Mr. D. M., æt. 23 years, of good constitution and sanguine temperament; found him with a general exaltation of the circulation, pulse quick and rather sharp than strong, tongue red and somewhat furred. Proposed v. s. to which he objected, if it could be dispensed with. Prescribed calomel, ipecacuanha and pulv. antimonialis every four hours.

15*th.* Visit repeated. Good bilious purging had been procured, and case seemed better. Prescription continued.

16*th.* Patient up; ceased to take medicine.

19*th.* Recalled; found a recurrence of first symptoms increased in intensity; prescribed calomel and ipecacuanha. For some days the case did not materially change; but after a sudden and severe alteration in the weather, which was not counteracted by a corresponding addition to the bed clothing, pneumonic symptoms were superadded. These were speedily subdued by cupping and blisters, and the case went on as before, except that there were irregular chills occurring once in three or four days. On the 25th the green mucous evacuations appeared, with cool skin, small, weak, and quick pulse; intellect unimpaired. Prescribed calomel and sulph. quin. freely, with blisters to the stomach, wrists and ankles.

29*th.* Feeble reaction.

30*th.* Death closed the scene.

*Case* 2*d.* June 21st, 1838, I was requested to see Mrs. M., æt. about 20 years, of delicate constitution, who had, six weeks prior to this date, given birth to her second child. Symptoms; slight acceleration of the circulation, some dryness of the tongue, and constipation of the bowels. Prescription; mild cathartic pills, which gave so much relief, that the patient was up the greater part of the succeeding day.

23*d*, Recalled. Symptoms: small, weak, and frequent pulse, cool skin, dry brown tongue, pulmonary oppression. As I had not been the regular family physician, I apprized the husband of my opinion, that it would be a dangerous case, and suggested the propriety of calling an additional medical attendant. Two others were called, and for two or three days, sinapisms, blisters, calomel, quinine, carb. ammonia, seneka, and other articles were used, according to the indications of the case. The disease progressed; green mucous discharges speedily appeared; neither reaction nor secretion could be induced; two or three days after the appearance of the green mucus, gangrenous spots appeared on the gums, and under the tongue, from which there was considerable hæmorrhage. The alvine dejections continued unchanged to the death of the patient, which occurred on the 5th of July. In this case, also, the intellect continued unimpaired to the end.

*Case* 3d. July 19th, 1838—called to see Catharine, a servant, æt. 16 years, robust and fully grown. This girl had waited on the lady whose case has just been detailed. Symptoms similar to those in that case, but more violent. Calomel, ipecacuan, and rhubarb were prescribed.

21*st.* Green mucous discharges; 23d, gangrenous and hæmorrhagic gums; green mucus continued to be discharged; general coldness of the surface; very small, weak, and frequent pulse; great pulmonary oppression. This was the fifth case of the same character, that I had seen, all of which had a fatal termination. I was unwilling that any more should die under the same treatment, although, *a priori*, I considered it the best that I could institute. I was satisfied that the pathology did not consist in portal congestion, or gastro-intestinal inflammation; and I recollected that in the winter 1832, '33, Professor Caldwell had suggested it as probable, that the reason why calomel did not readily excite the liver in cholera, was, that the ganglionic nerves supplying the liver were so oppressed by disease, that they did not convey to that organ its proper amount of nervous energy, and that, as a consequence, it was not susceptible to the impression of stimuli. If this pathological view was correct in cholera, and I thought it probable, the same condition might have obtained in congestive fever; and as the ganglia proceed from spinal radicles, this new view of its pathology suggested the propriety of strong stimulation to the spine, with the intention of arousing the nervous influence, and thus transmitting it to the organs requiring it. The gangrene of the mouth forbade the further use of mercurials, and under this aspect of the case, the following prescription was made: sinapisms to the whole length of the spine, and pills of rhubarb. In twelve hours there was general reaction, and in twenty-four, the alvine dejections consisted of a mixture of mucus and bile. Subsequent treatment—sinapisms re-applied every twelve hours, and the bowels gently evacuated twice or thrice in the same time with rhubarb. Under this treatment the case slowly, though steadily, recovered.

*Case 4th.* Before my attendance on the last case was discontinued, Lucy, a negro woman in the same family, aged 25 years, was attacked with symptoms similar to those in it. A dose of calomel was administered, and once repeated; under the operation of the second dose, the green mucus discharges appeared. Sinapisms, as in the other case, were applied to the whole length of the spine every twelve hours, and the bowels evacuated with rhubarb. Under this treatment the patient speedily recovered.

*Case 5th.* Chaney, aged 12 years, a servant girl, belonging to the same family, being similarly attacked, a dose of calomel was administered which produced green mucus discharges, and dry, black gums. Sinapisms and rhubarb were used, as before, with the happiest consequences—the improvement was perceptible in twelve hours, and continued steadily to perfect recovery.

*Case 6th.* September 1st, 1838, I was requested to visit a youth, aged 12 or 13 years, of good constitution, who had been sick some twelve days. Symptoms—expressed himself as being free from pain; tongue dark red and very dry; skin rather hot; pulse too frequent and sufficiently full; restlessness very considerable. He had been in a similar condition to that just stated, from the commencement of the attack; had taken 25 grs. calomel every night, with rhubarb on the following morning, when the calomel failed to operate. Discharges represented as having been consistent and bilious from the beginning. The same treatment was continued for one night longer.

*Sept. 3d.* Gangrene of the gums apparent; surface cool; pulse small, weak and frequent; alvine evacuations represented as being still consistent and bilious; but upon inspection were found to be a dark green mucus. Spinal irritation with mustard seed was used every twelve hours, and the bowels moved with rhubarb. Under this treatment the patient recovered, but was considerably endangered for some days by gangrene and hæmorrhage of the gums.

*Case 7th.* September 8th, 1838, was requested to consult in the case of a child aged 3 years, who had been sick fourteen days; disease supposed to be worms; between twelve and twenty doses of calomel had been given, and pink root in proportion. Present symptoms—cool surface; small, weak and frequent pulse; green mucous discharges; gangrene and ulceration of the gums, and of superior maxillary bone, beginning at the incisores, and extending backward along the palate bones, denuding them in their course, nearly to the velum pendulum palati. Treatment.—Sinapisms to the spine, with calcined magnesia and charcoal every two hours; sulph. cupri to the ulcers. The effect of this treatment was almost magical. In twenty-four hours the discharges were healthy; appetite good; reaction perfect, and every vestige of disease removed, except the gangrene of the mouth. This unfortunately continued, removing several teeth, destroying the upper lip and nose, and extending (before death, which did not occur for nearly three weeks,) to the forehead. During the greater part of this time, the general strength improved; the appetite was excellent, and the evacuations were natural.

Since the occurrence of the above cases, it has fallen to the lot of the writer to witness a vast number of others bearing a strong family likeness to them, but presenting great varieties in some of their features. The years of 1839 and 1840 have ben remarkably prolific in such forms of disease, investing nearly all febrile affections with their liveries. In the cases narrated no regular, nor indeed irregular, remissions were observed; whereas, in the years 1839 and 1840, the chief part might with great propriety be denominated congestive remitting fever, and almost every medical practitioner in southern Kentucky, would under that term recognize their unwelcome annual visitant.

It is not my purpose to go into a formal detail of any other cases, but merely to give a succinct synopsis of my observations on the pathology and treatment of them.

My investigations have led me to the following conclusions, viz; that the disease consists, first in an oppression of the nervous system, and, as an unavoidable consequence, the equilibrium in the circulation is destroyed; that either of the above conditions is most certainly productive of diminished or arrested secretion, and great visceral congestions, which are very prone to run into sluggish inflammations and gangrene; that the green mucous discharges observed are not "*green gelatinous bile*", but a vitiated acid secretion from the intestinal mucous membrane, which results in ulceration; and that the predisposition in this form of fever to vitiated acid secretion is excited, and kept in destructive activity, by mercurials.

In all cases of ordinary violence, the respiration is very laborious, being performed, in a great measure, by the elevation of the shoulders, and failing to reach the bottom of the lungs, which renders it nescessary, every few minutes, to make a deep inspiration. During this state of the respiratory apparatus, there is an almost intolerable sensation of weight at the epigastrium, with feeble radial pulsation, and cool surface, especially of the extremities. In this condition, I have endeavoured to arouse and equalize the circulation by the application of sinapisms, together with warm rocks to the extremities, without making the slightest impression, or producing the least redness under the mustard plasters. In the same cases and conditions, I have applied the counter-irritant to the whole length of the spine, and invariably with the most marked advantage. It has always aroused the circulation, warmed the extremities, removed the epigastric oppression, and often induced a warm, pleasant diaphoresis. In these cases, the tongue is often dry, brown in the centre, and fiery around the edges; the stomach is intolerant of pressure, and the whole group of symptoms forbids the internal exhibition of stimulants.

I have seen several cases where instead of mustard seed, blisters were applied to the spine, and with good results; but I prefer the mustard, because the intensity of its effects can be increased or mitigated at pleasure; and therefore the spinal cord does not become so accustomed to its stimulation as it does to that of a more permanent counter-irritant; and also because it is exceedingly difficult, not to say impossible, to confine the necessary dressings to a blistered spine.

I have said that a great number of these cases have remissions, and I may add that they are more difficult to manage on account of these remissions. A case may have existed and been under treatment for several days, or a week, and may seem to be doing well, when suddenly the voice will sink and become a husky whisper; the end of the nose and sometimes the extremities of the fingers and toes become cool, while the rest of the body evinces preternatural heat; the patient complains of a sensation of sinking, with alternations of hot flashes and slight rigors. This condition not unfrequently continues for four or six hours with great intensity, and it may require twenty four or thirty six hours for reaction to be as well established as before the paroxysm. In a few cases death takes place in the first paroxysm; and if not arrested would usually do so in the second or third.

How are these paroxysms to be prevented? This is a question of great difficulty. The dry, reddish brown tongue, the great epigastric tenderness and heat, have always seemed to me plainly to contra-indicate the use of sulphate of quinine; and yet a recurrence of the paroxysm must be obviated, or death is inevitable. Under these circumstances I have given, several hours before the accession of the coolness was anticipated, 10 gr. pulv. Doveri, 5 gr. camphor, with a cup of hot sage or horsemint tea, and applied sinapisms to the spine and extremities, together with warm rocks to the hands and feet; and this practice has not disappointed me. It has uniformly produced the intended effect. I have not depended on spinal irritation alone to arrest these paroxysms, because the danger is so imminent, that I have felt disposed to employ every adjuvant within my reach. After arresting it once, the same means to a less extent should be subsequently used as the period of recurrence approaches.

*January*, 1841.

